# mTOR Inhibition by Everolimus in Childhood Acute Lymphoblastic Leukemia Induces Caspase-Independent Cell Death

**DOI:** 10.1371/journal.pone.0102494

**Published:** 2014-07-11

**Authors:** Rana Baraz, Adam Cisterne, Philip O. Saunders, John Hewson, Marilyn Thien, Jocelyn Weiss, Jordan Basnett, Kenneth F. Bradstock, Linda J. Bendall

**Affiliations:** 1 Centre for Cancer Research, Westmead Millennium Institute, University of Sydney, Westmead, NSW, Australia; 2 Department of Haematology, Westmead Hospital, Westmead, NSW, Australia; Roswell Park Cancer Institute, United States of America

## Abstract

Increasingly, anti-cancer medications are being reported to induce cell death mechanisms other than apoptosis. Activating alternate death mechanisms introduces the potential to kill cells that have defects in their apoptotic machinery, as is commonly observed in cancer cells, including in hematological malignancies. We, and others, have previously reported that the mTOR inhibitor everolimus has pre-clinical efficacy and induces caspase-independent cell death in acute lymphoblastic leukemia cells. Furthermore, everolimus is currently in clinical trial for acute lymphoblastic leukemia. Here we characterize the death mechanism activated by everolimus in acute lymphoblastic leukemia cells. We find that cell death is caspase-independent and lacks the morphology associated with apoptosis. Although mitochondrial depolarization is an early event, permeabilization of the outer mitochondrial membrane only occurs after cell death has occurred. While morphological and biochemical evidence shows that autophagy is clearly present it is not responsible for the observed cell death. There are a number of features consistent with paraptosis including morphology, caspase-independence, and the requirement for new protein synthesis. However in contrast to some reports of paraptosis, the activation of JNK signaling was not required for everolimus-induced cell death. Overall in acute lymphoblastic leukemia cells everolimus induces a cell death that resembles paraptosis.

## Introduction

The paradigm of how anti-cancer treatments kill cancer cells has been that these agents induce apoptotic cell death, and cells with defective apoptotic machinery are therefore resistant to therapy. However this model does not align well with clinical experience [1]. Increasing numbers of reports describing non-apoptotic death mechanisms elicited by a range of current and potential anti-cancer agents have emerged over the last decade [2]–[4]. Currently non-apoptotic cell death has been classified into several categories. However the exact mechanisms involved are not well defined and it appears that a myriad of related and overlapping death mechanisms exist. Some of the better-recognized forms of non-apoptotic cell death include: Type II cell death or autophagic cell death, Type III cell death or paraptosis, mitotic catastrophe, and necroptosis, a form of regulated necrosis. Essentially all non-apoptotic cell death mechanisms lack many of the features of apoptosis including chromatin condensation, DNA fragmentation, caspase activation and membrane blebbing [5], [6].

Type II cell death is regulated by a highly conserved group of autophagy-related genes and is characterized by the accumulation of double-membrane-bound vesicles called autophagosomes. These fuse with lysosomes resulting in the degradation of their contents. However, autophagy is perhaps better known as a cell survival mechanism, removing damaged organelles and providing recycled nutrients [7]. Necroptosis is a type of programmed necrosis that can occur when apoptosis is blocked while events that normally induce apoptosis are activated. The morphological features of necroptosis are typical of pathological necrosis and include organelle swelling, rapid mitochondrial dysfunction, plasma membrane permeabilization and lack of nuclear fragmentation [8]. Mitotic catastrophe results from mitotic failure caused by defective cell cycle checkpoints, disruption of microtubule regulation and/or DNA damage. Caspases may be activated but it is generally independent of caspase activity [9]. The main features of Type III cell death or paraptosis are extensive cytoplasmic vacuolization and swelling of endoplasmic reticulum (ER) and/or mitochondria [10]. It has been variably reported to be dependent or independent of protein synthesis and MAPK signaling [11]–[13].

We have previously reported that mTOR inhibition by everolimus results in a non-apoptotic cell death in human ALL cells in a NOD/SCID mouse model of human ALL [14]. This finding was largely based on morphology, the delayed cleavage of PARP and the presence of LC3 processing. However details of the death mechanism were not further explored. In this manuscript we have further characterized the mechanism underlying cell death induced by mTOR inhibition in ALL cells using *in vitro* systems. We find that cell death is not apoptotic in nature and although a strong autophagic response is evident, inhibition of autophagy does not prevent cell death. Despite JNK activation, cell death was not dependent on MAPK signaling, however new protein synthesis was required. The induction of ER stress was present although the induction of genes encoding for chaperones was strongly suppressed, with changes in protein levels being confined to HSP70. The cell death induced by everolimus in ALL cells resembles paraptosis although there are differences in our observations and the standard definition of this death mechanism.

## Materials and Methods

### Cells

Human precursor-B ALL cell lines were obtained as follows: NALM6 from Deutsche Sammlung Von Mikroorganismen und Zellkulturen Gmbh (ACC-128); Reh from American Type Culture Collection ATCC (CRL-8286); and LK-63 were a gift from Professor Andrew Boyd (Queensland Institute of Medical Research, Brisbane, QLD, Australia) [15]. Cells were maintained in RPMI medium containing 10% fetal calf serum (FCS) (complete media) as previously described [16]. Patient samples were obtained following informed written consent with Sydney West Area Health Service Human Research Ethics Committee approval (Sydney, NSW, Australia). Details of patient samples have been previously published and are provided in Table S1 [17]. Cells from all samples except 1901 were expanded on stroma to provide sufficient material for the study.

### Antibodies and Reagents

Everolimus was kindly provided by Novartis (Basel, Switzerland). Caspase-8 (BF4100) and caspase-9 (BF10100) activity assays were purchased from Bioscientific. The pan-caspase inhibitor ZVAD-FMK, 7AAD, 10× annexin V binding buffer, 10× perm/wash buffer, annexin V-FITC (556419) and streptavidin APC (554067) were purchased from Becton Dickinson. 3-Methyladenine (3MA) was purchased from Sigma Aldrich (M9281), Tetramethyl rhodamine methyl ester (TMRM, T668) and Annexin V alexa fluor 647 (A23204) were obtained from Invitrogen. The JNK Inhibitor SP600125 (420119) and InnoCyte Flow Cytometric Cytochrome c Release Kit (CBA077) were purchased from Merck. The following antibodies to human antigens were purchased: mouse active caspase-3 FITC (559341) or biotin (550537) and PARP (556362) (Becton Dickinson); rabbit anti-c-Jun phosphorylated on Ser^63^ (9261), poly (ADP-ribose) polymerase 1 (PARP) (9542), S6 ribosomal protein (S6RP) (2217), S6RP phosphorylated on Ser^235/236^ (4856), eukaryotic translation initiation factor 4E binding protein 1 (4E-BP1) phosphorylated on Thr^37/46^ (2855), microtubule-associated protein 1 light chain 3 alpha (LC3) (2775), heat shock 70 kDa protein 5 (BiP) (3183), eukaryotic translation initiation factor 2, subunit 1 alpha (9722), 35 kDa (eIF2α) phosphorylated on Ser^51^ (9721), heat shock 60 kDa protein 1 (HSP60) (4870), heat shock 70 kDa protein (HSP70) (4872), and heat shock 90 kDa protein (HSP90) (4877), heat shock transcription factor 1 (HSF1) (4356), (Cell Signaling Technologies) CHOP (NB100-91777, Sapphire Bioscience). Z-VAD (550377) Becton Dickinson, cyclohexamide (C-7698, Sigma-Aldrich), β-actin clone AC74 (A2228, Sigma-Aldrich), Swine anti-Rabbit HRP secondary antibody (P021702) Dako Australia.

### Clonogenic Assays

ALL cells were exposed to indicated concentrations of everolimus for 16 hours in RPMI contain 10% fetal calf serum and then washed into drug free medium prior to being plating at 1 cell/well in 96 well plates. Wells were scored as positive or negative for cell growth after 3 weeks of culture. The proportion of positive wells was calculated and the values normalized to untreated controls.

### Flow Cytometry

Viability was assessed using annexin V and 7AAD staining as previously described, except that the propidium iodide was replaced with 0.25 µg/ml 7AAD [16]. Intracellular staining was performed on cells fixed in ice cold 70% ethanol and blocked in perm/wash buffer containing 10% human AB serum for 1 hour. Cells were labeled for 1 hour with appropriate primary antibodies, or isotype control antibody, for 1 h at room temperature in the dark. Cells were subsequently washed and resuspended in 500 µL PBS for analysis by flow cytometry. If required cells were incubated with species specific, fluorophore conjugated secondary antibodies for 45 min in the dark at room temperature. Cells were analyzed by flow cytometry using a FACSCalibur flow cytometer.

### Western Blotting

Cell lysates were prepared from cell pellets and equal amounts of protein loaded in each lane of 7.5 or 15% SDS-PAGE gels and transferred onto nitrocellulose membranes as previously described [18]. Phosphorylated and total proteins were detected sequentially on the same membrane using specific primary antibodies, appropriate secondary antibodies conjugated to HRP and enhanced chemiluminescence (NEL105, Perkin-Elmer). Bands were quantitated by densitometry (Molecular Dynamics) using CareStream MI SE software.

### Electron Microscopy

Cell pellets were fixed in modified Karnovsky's fixative (2.5% formaldehyde prepared freshly from paraformaldehyde; 2.5% EM grade glutaraldehyde in 0.1M MOPS (3-[N-Morpholino]propanesulphonic acid) buffer, pH 7.4) then decalcified in 0.5M EDTA for 14 days. Tissue blocks were selected, trimmed and post fixed in osmium tetroxide, dehydrated in increasing concentrations of ethanol and embedded in epoxy resin. Semi-thin (500 nm) sections were cut on a Reichert ultracut microtome and assessed by light microscopy. Ultrathin (80–90 nm) sections were then cut and grid stained with 2% ethanolic uranyl acetate, then Reynolds lead citrate. The ultrastructure was examined using a Philips CM-10 transmission electron microscope operated at 80 KV. Images were recorded using Kodak electron microscope film type 4489. Black and white prints were scanned using an HP scanjet flatbed scanner and composite images compiled using Adobe Photoshop (Version 8) software.

### Microarray and Quantitative RT-PCR

Microarray was performed using the Illumina BeadArray Protocol. Briefly, RNA samples were reverse transcribed, amplified and labeled using the Illumina Amplification Kit (Invitrogen, AMIL1791). Samples were arrayed on the HumanRef-8 Sentrix BeadChip (BD-102-0203, BD-102-0603, Illumina), which allows genome-wide expression analysis, and data analyzed using the Illumina BeadStudio Gene Expression Module (Version 3) Software as per the manufacturers' instructions. Gene Expression signal levels were quantified using Gene Analysis module and a significance level of p<0.05. Differential expressions between paired samples were performed using the Differential Analysis module and a significance level of p<0.05. Gene set analysis was performed using Metacore Pathway Analysis software (Thomson Reuters) and DAVID Bioinformatics Resources 6.7 from the NIH (http://david.abcc.ncifcrf.gov).

Total RNA was extracted using Trizol reagent (15596-018, Invitrogen) and genomic DNA removed by RNase free DNase (M610A, Promega). RNA was reverse transcribed using MMLV Reverse Transcriptase (M170B, Promega) and oligo-dT primers (C110A, Promega) for 60 min at 37°C. The gene expression levels were quantitated from standard curves generated using cDNA prepared from NALM6 cells by real-time RT-PCR, using QuantiTect SYBR Green RT-PCR kit (204243, QIAGEN) and a Rotor-Gene 3000 thermocycler, as described previously [19]. The cycle threshold (Ct) was determined using standard curves in the Rotor Gene software. The relative expression ratio of the different target genes was compared with the housekeeping gene glyceraldehyde-3-phosphate dehydrogenase (GAPDH) using the same cDNA to generate the standard curve. Values were then normalised to the untreated sample. All samples were run in quadruplicates. Details of primers are given in Table S2.

### Statistics

Comparisons between two groups were performed using the Student's t test and between multiple groups using ANOVA analysis. A level of significance of <0.05 was deemed significant. The significance values for the microarray data shown have been corrected for multiple comparisons using the Benjamini method.

## Results

### Inhibition of mTOR results in caspase-independent ALL cell death

**Figure 1:**
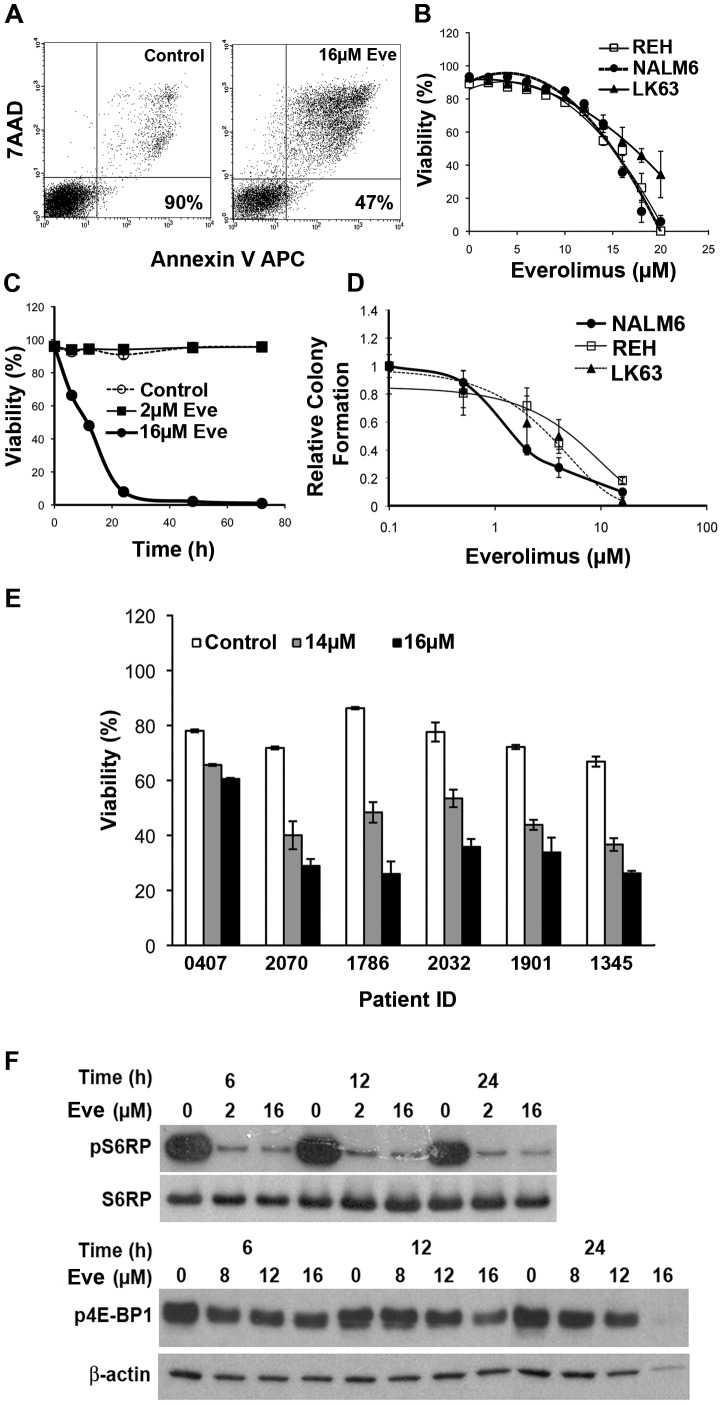
Everolimus induces a dose dependent cell death in ALL cell lines and patient samples. (**A**) Representative dot plots showing the staining with Annexin V and 7AAD on ALL cells treated with vehicle, or 16 µM everolimus for 24 h with the viability indicated on the plots. (**B**) Dose response curves for ALL cells lines following a 24 h exposure to the indicated concentrations of everolimus. (**C**) The viability of NALM6 cells treated with everolimus for the indicated time periods. (**D**) Clonogenic potential of the indicated ALL cell lines following overnight exposure to the indicated concentrations of everolimus. Data has been normalized to control cultures. (**E**) The effect of a 24 h exposure to the indicated concentrations of everolimus 1 on the viability of patient ALL samples. (**F**) Western blots showing the phosphorylation of S6RP and 4E-BP1 in NALM6 cells following treatment with indicated concentrations of everolimus over various time intervals.

The effect of the mTOR inhibitor everolimus on the survival of ALL cell lines was determined using annexin V and 7AAD staining and flow cytometry (Figure 1A). Cells became annexin V and 7AAD positive, demonstrating loss of viability with an IC_50_ of between 15 and 17 µM (Figure 1B). Lower concentrations of everolimus failed to induce cell death at time points up to 72 h (Figure 1C). However, overnight exposure to lower concentrations of everolimus had a greater effect on the clonogenic survival of ALL cells with IC_50_ values of 1.5, 4 and 3.5 (Figure 1D) for the three cell lines tested. While this could be due to induction of senescence there was no evidence of β-galactosidase expression in everolimus treated cells even after 14 days (Figure S1) and the ALL cell lines used here lack p16 making senescence less likely (Figure S2). However this does suggest that a proportion of cells exposed to lower concentrations of everolimus are rendered incapable of further growth, despite not being assessed as senescent by β-galactosidase expression or non-viable by annexin V and 7AAD staining. Patient samples that had been expanded *in vitro* in stromal dependent cultures, and one previously uncultured sample (1901), demonstrated a similar sensitivity to everolimus as the cell lines (Figure 1E) in short term cultures. Inhibition of mTOR signaling was apparent with a dramatic inhibition of phosphorylation of S6RP and a smaller but significant suppression of phosphorylation of 4E-BP1 (Figure 1F). This data shows that everolimus induces cell death that is not associated with the induction of senescence.

**Figure 2:**
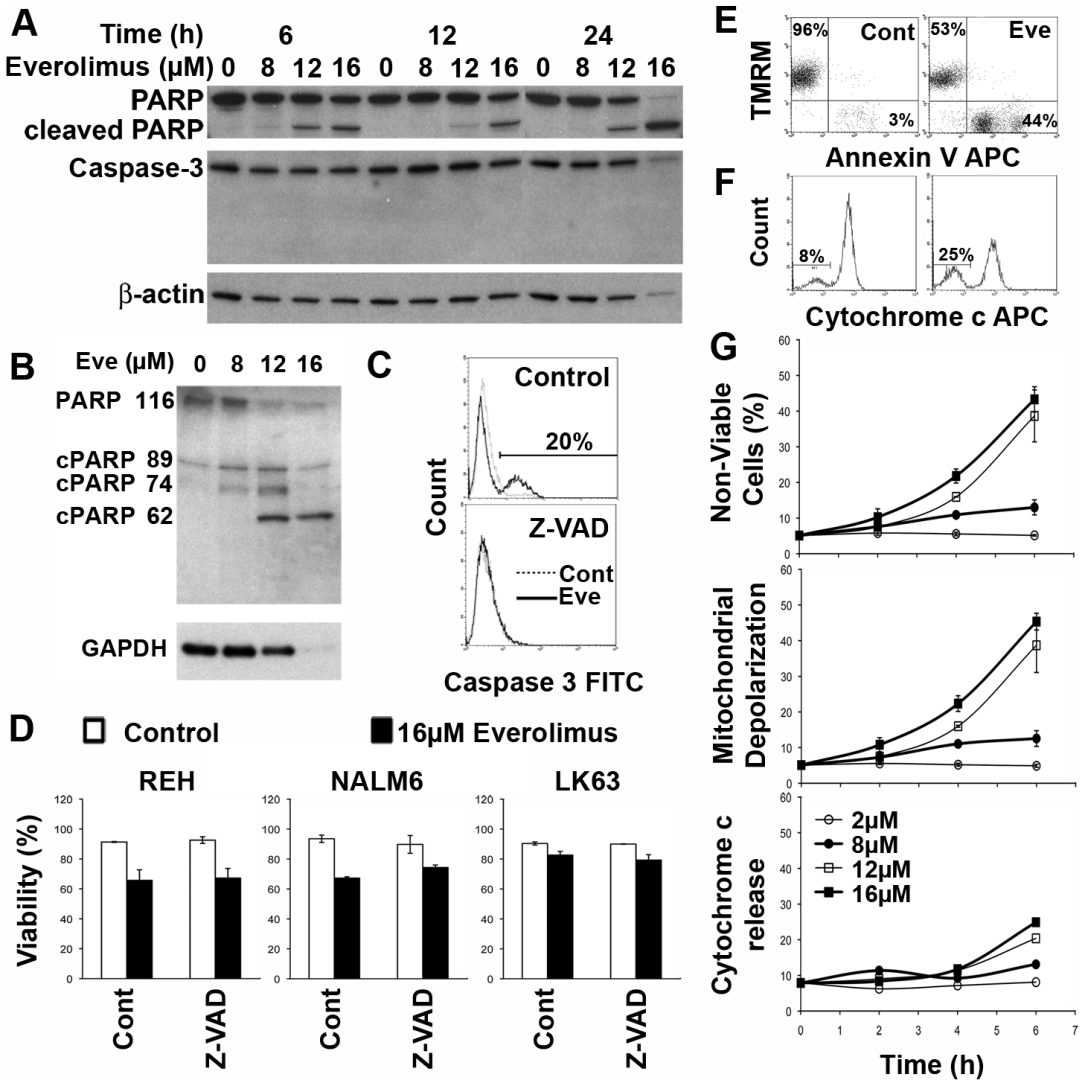
Everolimus induces a caspase independent cell death in ALL cells. **(A)** NALM6 cells were treated with indicated concentrations of everolimus for the specified time intervals and cell lysates prepared. Western blots for PARP and caspase 3 are shown with b-actin acting as a loading control. **(B)** NALM6 cells were treated with indicated concentrations of everolimus for 24 h and cell lysates prepared. Western blots for PARP using the C2-10 antibody are shown with b-actin acting as a loading control. **(C)** NALM6 cells were pretreated with Z-VAD (right panel) or vehicle (left panel) and then exposed to 16 µM everolimus or placebo for 24 hours. Cells were stained for activated caspase 3 and analyzed by flow cytometry and overlay histograms of placebo and everolimus treated cells is shown. **(D)** The indicated cell lines were treated as in (B) were labeled with Annexin V and 7AAD and analysed by flow cytometry. The percentage of viable cells (dual negative cells) has been plotted. **(E)** NALM6 cells were treated with placebo or 16 µM everolimus for 6 hours and then stained using TMRM and annexin V. Representative flow plots are shown. The percentage of TMRM positive/annexin V negative and TMRM negative/annexin V positive cells are shown in the upper left and lower right quadrants respectively. **(F)** Cells treated as in D were stained for cytochrome c. The percentage of cells negative for cytochrome c is shown. **(G)** NALM6 cells treated as above for the indicated time periods with the specified concentrations of everolimus were assessed by flow cytometry for viability (upper panel), mitochondrial depolarization (middle panel) or cytochrome c release (lower panel) by flow cytometry as described for C, D and E respectively.

While cells undergoing cell death as a result of exposure to everolimus bound annexin V, they were also positive for 7AAD staining, suggesting that annexin V binding could be the result of loss of plasma membrane integrity. Western blotting revealed that some PARP cleavage could be detected in everolimus treated cells and this occurred in a concentration and time dependent manner (Figure 2A). In addition to the apoptosis and necrosis-induced 89 kDa fragment, the necrosis associated 74 and 62 kDa fragments were more prominent, suggesting that apoptosis was not the primary death mechanism (Figure 2B) [20]. However we were unable to detect cleavage of the principal executioner caspase, caspase-3 (Figure 2A), although we could detect caspase-3 cleavage following treatment with doxorubicin (Figure S3). Using flow cytometry, low levels of cleaved caspase-3 were detected and could be completely inhibited by pre-incubation with the pan-caspase inhibitor Z-VAD-FMK (Figure 2C). Although the activity of caspase-8 and -9 were both increased (Figure S4), the addition of Z-VAD-FMK failed to prevent the induction of cell death by everolimus, suggesting that despite some caspase activation, cell death was caspase independent (Figure 2D). Short time points have been shown in an attempt to maximize the possibility of inhibiting cell death by minimizing the opportunity for the subsequent induction of alternative death mechanisms in the face of caspase inhibition, however similar results were obtained at later times when cell death was more extensive.

Mitochondrial depolarization (ΔΨ_m_) is associated with apoptotic cell death, but has also been demonstrated in other forms of cell death [21]. Everolimus induced mitochondrial depolarization in a dose dependent manner, which was tightly associated with loss of cell viability (Figure 2E & F). In contrast, release of cytochrome c occurred in a smaller percentage of cells and at later time points than mitochondrial depolarization and cell death (Figure 2G). This suggests that cytochrome c release is not responsible for, but is a consequence of, the observed cell death, consistent with caspase independence.

### Inhibition of mTOR results in the induction of autophagy

**Figure 3:**
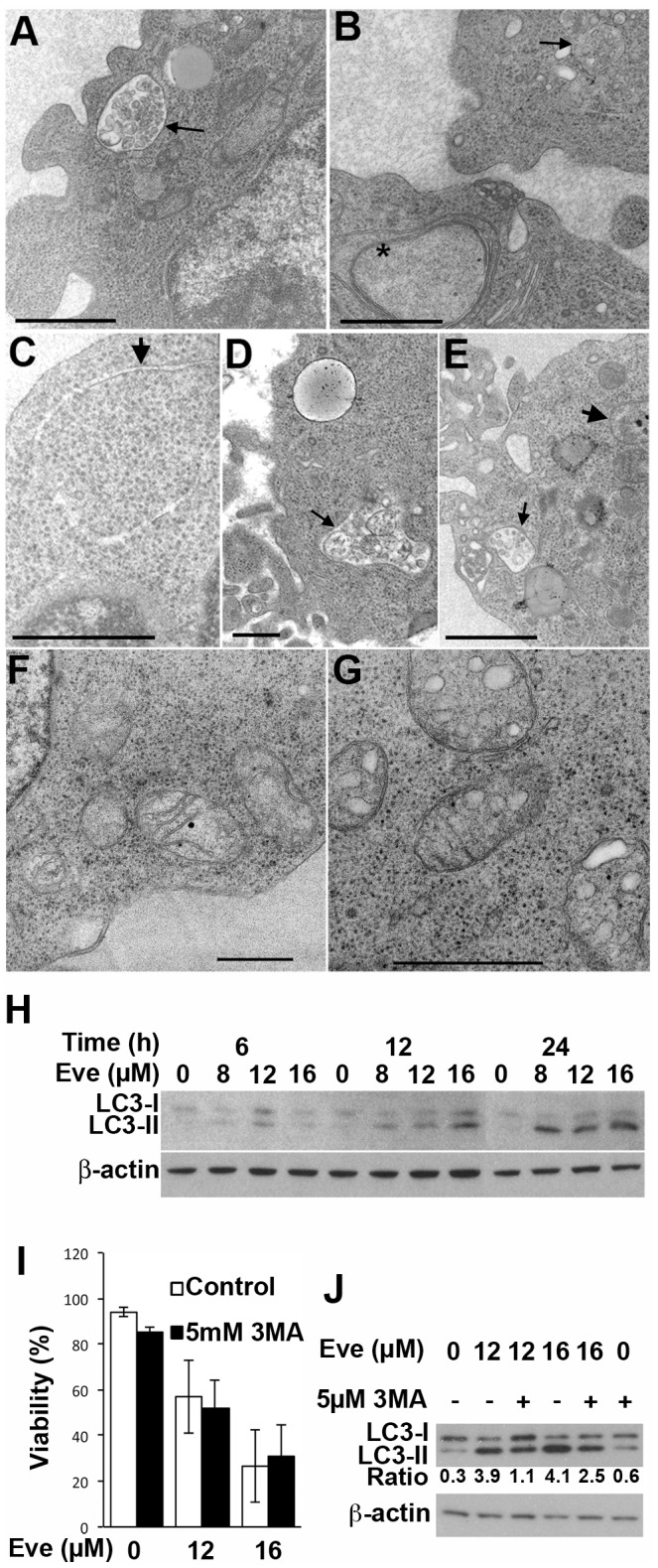
Everolimus-Induced Autophagy is Not Required for Cell Death. (**A–G**) NALM6 cells were treated with vehicle or 16 µM everolimus overnight and ultrastructure examined by electron microscopy. Fine arrows indicate autophagic vacuoles containing degrading organelles. Thick arrows indicate stretches of double membrane walling off regions of cytoplasm. Scale bars indicate 100 nm. (**H**) Cell lysates prepared from NALM6 cells cultured for the indicated time periods with the specified concentration of everolimus were analyzed for LC3-I and LC3-II by Western blotting and sequential blotting for β-actin used as a loading control. (**I**) The viability of NALM6 cells cultured for 24 h with the indicated concentration of everolimus was assessed with and without the addition of 3MA. The mean ± SD of 3 independent experiments is shown and analyzed using a paired t-test. (**J**) Analysis of LC3 by Western blotting following a 24 h incubation of NALM6 cells with indicated concentration of everolimus with or without that addition of 3MA. The ratio of LC3-II to LC3-I is indicated below the blot.

We have previously reported that everolimus induces autophagy as determined by electron microscopy in human ALL cells in NOD/SCID mice but considered that apoptotic cells may have been rapidly removed in vivo and so went undetected [14]. However, consistent with the *in vivo* findings, ALL cell lines treated with 16 µM everolimus *in vitro* for 24 h also display features of autophagy with a paucity of cells having morphological features consistent with apoptosis (Figure 3A–E). We also previously demonstrated that everolimus induced acidic vacuoles, beclin-1 expression and LC3 processing following in vitro exposure to everolimus [14]. Here we confirm the dose and time dependence of LC3 processing in response to everolimus (Figure 3H). However inhibition of autophagy using 3MA did not prevent cell death despite the inhibition of LC3-II generation suggesting that autophagy was not the mechanism of death (Figure 3I & J). The morphological features we observed by electron microscopy included vesiculation, particularly swelling of the endoplasmic reticulum, with mitochondrial swelling being present on occasions. (Figure 3E & F) are also consistent with paraptosis [5].

### Everolimus Activates the ER Stress Response

Microarray analysis of NALM6 cells treated with 16 µM everolimus for 24 h revealed an up regulation of only 73 genes while 234 were down regulated. Metacore analysis revealed over-representation of genes encoding for nuclear (23 up regulated and 108 down regulated, p = 1.379e^−7^), mitochondrial (7 upregulated and 57 down regulated, p = 1.600e^−16^) and endoplasmic reticulum (6 up regulated and 73 down regulated, p = 0.0117) proteins. Using DAVID Bioinformatics Resource, genes involved in a range of biosynthetic pathways and processes including lipid synthesis (p = 0.0059), fatty acid biosynthesis (p = 0.0109), glycolysis (p = 0.0108), ribosome biogenesis (p = 0.0137) and the proteasome (p = 0.0034) were down regulated providing a picture of cells undergoing a general shut down of metabolic activity. In keeping with the lack of apoptosis, genes associated with the induction of apoptosis were not over represented (p = 1.74). Using keywords in DAVID Bioinformatics Resource we found molecular chaperones and stress response genes to be the most enriched in the dataset suggesting that stress responses were playing a role (Table S3). The details of the genes in these classifications are given in Table 1.

**Table 1:** Molecular Chaperone and Stress Response Genes Regulated by Everolimus.

Gene Name	Description	Alternate Aliases	Fold Change
TRIB3	Tribbles homolog 3 (Drosophila) (TRIB3), mRNA.	RP5-1103G7.7, C20orf97, NIPK, SINK, SKIP3, TRB3	2.55
PPP1R15A	Protein phosphatase 1, regulatory (inhibitor) subunit 15A (PPP1R15A), mRNA.	GADD34	2.00
CCT8	Homo sapiens chaperonin containing TCP1, subunit 8 (theta) (CCT8), mRNA.	C21orf112, Cctq, D21S246, PRED71	−1.56
HSPE1	Heat shock 10 kDa protein 1 (chaperonin 10) (HSPE1), mRNA.	CPN10, GROES, HSP10	−1.57
TCP1	t-complex 1 (TCP1), transcript variant 1, mRNA.	CCT-α, CCT1, CCTa, D6S230E, TCP-1-α	−1.65
HSPA4	Heat shock 70 kDa protein 4 (HSPA4), transcript variant 1, mRNA.	APG-2, HS24/P52, MGC131852, RY, hsp70, hsp70RY	−1.68
HSPD1	Heat shock 60 kDa protein 1 (chaperonin) (HSPD1), nuclear gene encoding mitochondrial protein, transcript variant 1, mRNA.	CPN60, GROEL, HLD4, HSP60, HSP65, HuCHA60, SPG13	−1.72
HSPH1	Heat shock 105 kDa/110 kDa protein 1 (HSPH1), mRNA.	RP11-173P16.1, HSP105, HSP105A, HSP105B, KIAA0201, NY-CO-25	−1.75
CCT6A	Chaperonin containing TCP1, subunit 6A (zeta 1) (CCT6A), transcript variant 1, mRNA.	CCT-ζ, CCT-ζ-1, CCT6, Cctz, HTR3, MGC126214, MGC126215, MoDP-2, TCP-1-ζ, TCP20, TCPZ, TTCP20	−1.78
AHSA1	Activator of heat shock 90 kDa protein ATPase homolog 1	AHA1, C14orf3, p38	−2.00
HSP90AA1	Heat shock protein 90 kDa alpha (cytosolic), class A member 1 (HSP90AA1), transcript variant 2, mRNA.	HSP86, HSP89A, HSP90A, HSP90N, HSPC1, HSPCA, HSPN, Hsp89, Hsp90, LAP2	−2.19
HSPA8	Heat shock 70 kDa protein 8 (HSPA8), transcript variant 1, mRNA.	HSC54, HSC70, HSC71, HSP71, HSP73, HSPA10, LAP1, NIP71	−2.34
HSPA1A	Heat shock 70 kDa protein 1A (HSPA1A), mRNA.	HSP70-1, HSP70-1A, HSP70I, HSP72, HSPA1, HSPA1B	−4.99

**Figure 4:**
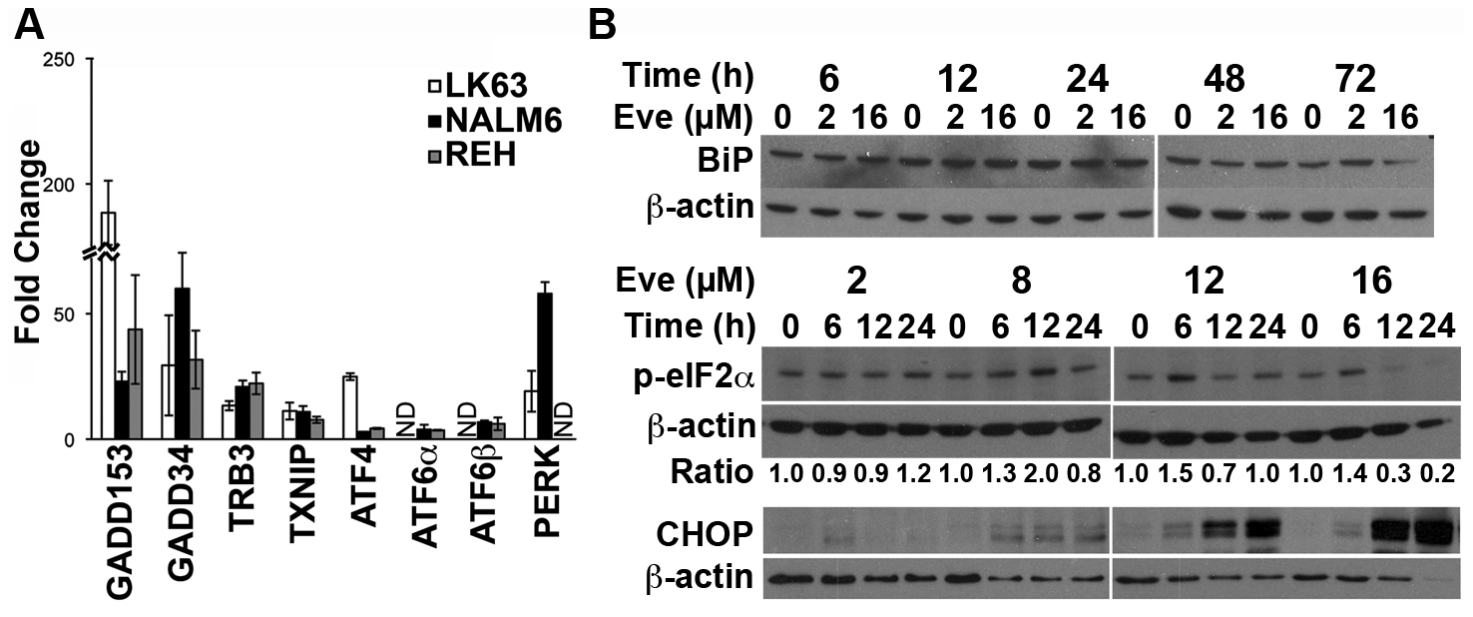
Everolimus Induces ER Stress. (**A**) NALM6 cells were treated with 16 µM everolimus overnight and qRT-PCR used to evaluate the expression of the indicated genes. The mean ± SD of the fold change from 3 experiments is shown. (**B**) NALM6 cells were incubated with the indicated concentrations of everolimus for the specified time periods and cell lysates prepared. Sequential Western blotting was performed on the same membrane to determine the levels of the indicated proteins in each series of panels. Phospho-eIF2α bands were quantitated by densitometry and normalized to β-actin.

The induction of ER stress genes was confirmed by quantitative RT-PCR (Figure 4A). Western blotting failed to detect any change in the expression of the most upstream component of the ER stress response, BiP (Figure 4B) and we were unable to detect processing of XBP1 at either the protein or mRNA level (data not shown). However, PERK mRNA was induced and eIF2α transiently phosphorylated at concentrations of everolimus greater than 2 µM. The timing of eIF2α phosphorylation was dose dependent with higher doses producing a more rapid response (Figure 4B). The phosphorylation of eIF2α was transient consistent with feedback inhibition of the pathway by the induction of GADD34 expression (Figure 4A) [22]. Increased ATF4 mRNA was detected in response to everolimus and expression of DDIT3/CHOP mRNA and protein also increased in a time and dose dependent manner. Overall everolimus treated cells display features consistent with the induction of endoplasmic reticulum stress.

### Cell Death Required New Protein Synthesis but was Independent of MAPK Signaling

**Figure 5:**
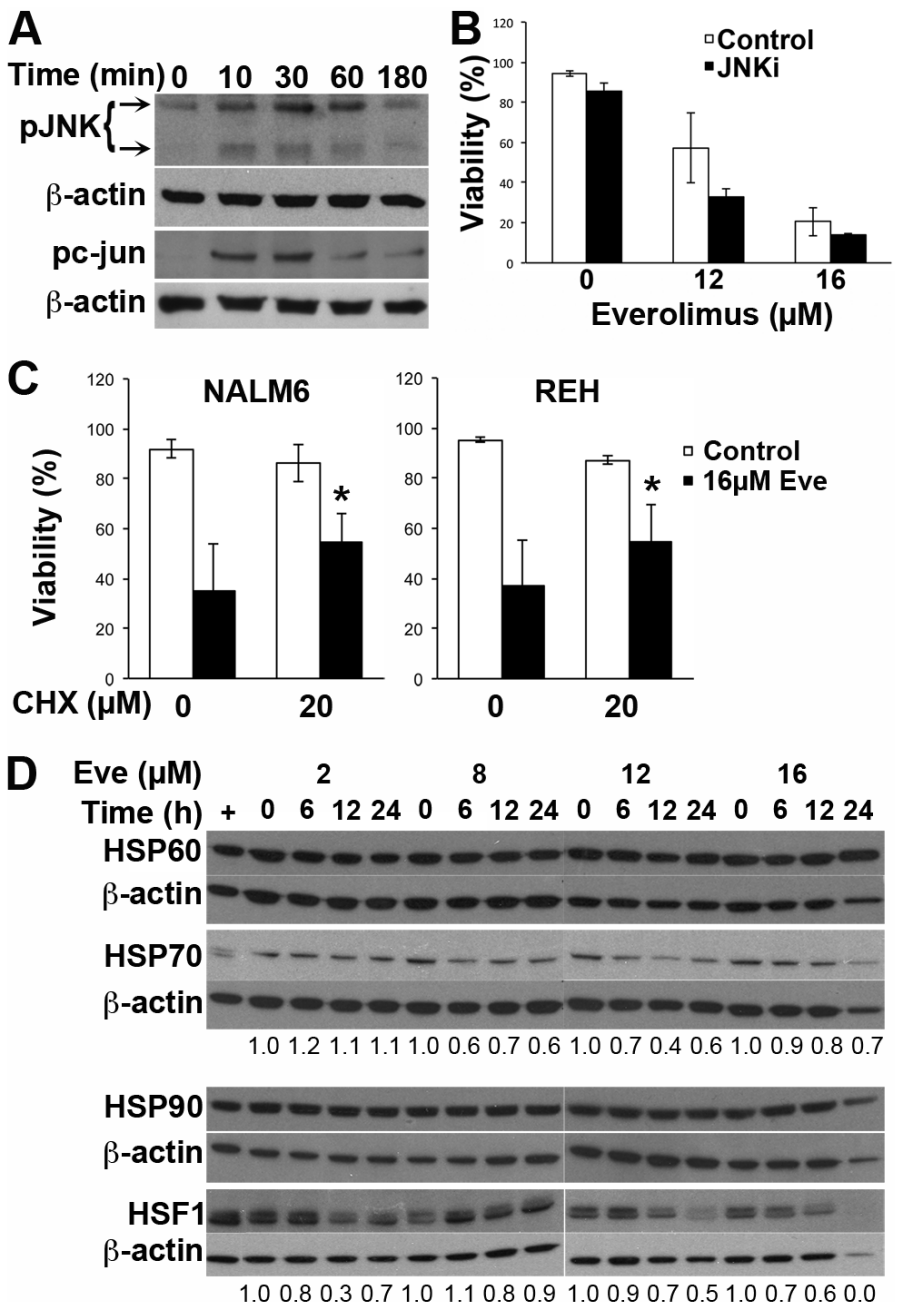
Cell death is dependent on new protein synthesis but not JNK signalling. (**A**) NALM6 cells were treated for the indicated time with 16 µM everolimus and cell lysates prepared. Lysates were probed for phosphorylated JNK (p-JNK) and loading assessed using β-actin. (**B**) NALM6 cells were treated with the indicated concentrations of everolimus for 16 h, with or without a 1 h pre-incubation with the JNK inhibitor SP600125 (5 µM) and viability assessed using annexin V/PI staining. The percentage of viable cells is shown with the bars representing the mean ± SE of 2 experiments. (**C**) NALM6 or REH cells were incubated with indicated concentrations of cyclohexamide for 1 h prior to the addition of the indicated concentration of everolimus. The mean ± SD of ≥3 independent experiments is shown. *p<0.05 when comparing cells with and without cyclohexamide. (**D**) NALM6 cells were treated with the indicated concentrations of everolimus for the specified times and cell lysates prepared. The indicated proteins were assessed by Western blotting and where indicated the bands were quantitated by densitometry using β-actin for normalization.

Inhibition of protein synthesis with cyclohexamide significantly reduced cell death (Figure 5C), demonstrating that everolimus-induced cell death is an active process and therefore a form of programmed cell death. Although JNK was rapidly activated following exposure to everolimus (Figure 5A), inhibition of JNK signaling (Figure S5) did not prevent cell death (Figure 5B). Microarray data revealed a reduction in the gene expression of molecular chaperones including heat shock proteins (Tables 1). HSF1, a transcription factor central to the expression of HSPs was decreased by everolimus treatment in a dose and time dependent manner. Although this translated into reduced HSP70 protein levels, HSP90 and HSP60 proteins were not measurably affected (Figure 5D and data not shown).

## Discussion

ALL is the most common childhood cancer and also affects adults with the majority of patients having disease originating in cells of the B lineage. The treatment of childhood ALL is one of the success stories of modern oncology [23], however approximately 15% of children and the majority of adults relapse following treatment. The overall survival of adults with relapsed disease is only 7% at 5 years [24], [25]. New strategies for the treatment of ALL are required to improve cure rates and reduce the toxicity of current treatment protocols.

The PI3K/Akt/mTOR signal transduction pathway is central to cell growth, proliferation and survival, and is implicated in ALL pathogenesis [26]–[28]. We, and others have previously reported pre-clinical data showing increased survival in xenograft models of human ALL [14], [29]. A completed clinical trial of the everolimus in non-Hodgkin's lymphoma patients demonstrated acceptable toxicity [30], and a current clinical trial of everolimus in combination with Hyper-CVAD chemotherapy [31] in patients with relapsed/refractory ALL has produced encouraging early data with the combination being well tolerated [32]. While it is unlikely that micro-molar plasma concentration of RAD001 can be achieved *in vivo*, preclinical studies have demonstrated *in vivo* efficacy at considerably lower plasma concentrations. An explanation for this discrepancy has not come to light and the potential for elevated location concentrations within crucial micro-environmental niches remains a possibility. These data support the possibility that everolimus or alternative mTOR inhibitors will be used in the treatment of ALL. However the mechanism by which everolimus induces cell death in ALL cells has not been explained.

Early reports on the effect of mTOR inhibition in ALL using the related mTOR inhibitors rapamycin and CCI-779 referred to the death mechanism as apoptosis, but annexin V staining was used in isolation, which is not sufficient to determine apoptotic cell death [29], [33]. Later studies were unable to demonstrate apoptosis as the principal death mechanism although a small subset of cells appeared to undergo apoptosis when mTOR was inhibited [17], [34]. In this study we evaluated the mechanism of cell death induced by mTOR inhibition and consistent with our previous reports, a small proportion of cells appear to undergo apoptosis, with very low levels of caspase-3 activation. However, apoptosis was absent from the majority of the dead and dying cells and caspase inhibition was unable to prevent cell death. Most interesting, was the discrepancy between cell death in short term *in vitro* cultures, which required very high concentrations of everolimus, and the inhibition of clonogenic cell growth which was affected by much lower concentrations of drug despite a shorter exposure time. This finding is consistent with the greater efficacy of everolimus in NOD/SCID xenograft models [14] than was expected based on *in vitro* studies [17] and suggests that standard assays for cell viability such as annexin V/PI staining underestimate the impact of everolimus on ALL cells.

The morphological features of cells exposed to everolimus are typical of autophagy, a well-known outcome of mTOR inhibition [35]. Biochemical data, showing lipidation of LC3 and accumulation of acid vacuoles, also support the induction of autophagy in these cells. However despite the clear induction of autophagy, inhibitors of this process failed to prevent cell death. The morphological features observed here are also consistent with paraptosis [10], and it is possible that both processes are occurring together as has been reported by others [13]. However, although swelling of the endoplasmic reticulum was observed, swelling of the mitochondria was only detected in a small proportion of cells. We have previously reported that everolimus induces cell death in cells committed to mitosis [36] but we did not observe dying mitotic cells. This is consistent with our previous report where the number of cells in mitosis was dramatically reduced by everolimus suggesting that death occurs in cells committed to undergoing mitosis but prior to chromosomal condensation and therefore is not classic mitotic catastrophe.

The requirement to commit to mitosis is also consistent with the difference observed with the short and long-term readouts of cell viability. This difference was not explained by replicative senescence, as the ALL cells studied here lack p16 and β-galactosidase was not induced. Furthermore, mTORC1 inhibition reportedly prevents senescence [37], [38]. It is possible that the slow kinetic observed here is due to the time taken for the cells to move through the cell cycle and commit to mitosis, a point where survival is not longer possible. We observed transient activation of JNK in ALL cells following mTOR inhibition. JNK activation is a known effect of mTOR inhibition with sustained activation reportedly required for apoptosis. The transient nature of JNK activation in ALL cells observed here could be a consequence of functional p53 in ALL cells [17], [39] as p53 prevents sustained activation of JNK and subsequent apoptosis [40]. This could explain why the everolimus-induced cell death in ALL cells is not mediated by JNK signaling.

New protein synthesis was required for cell death following exposure to everolimus, consistent with the activation of programmed cell death. This finding was perhaps surprising considering that inhibition of mTOR strongly suppresses CAP-dependent translation [41]. However CAP-independent (IRES-mediated) translation is activated in stressed cells and the proteins produced can modulate cell death [42]. Therefore it is possible that proteins translated in this manner are crucial for cell death in this setting, and that this CAP-independent translation is inhibited by cyclohexamide, which blocks elongation of all transcripts. We observed a reduction in HSP70 protein, although HSP60 and HSP90 proteins were not reduced despite decreased mRNA. The translation of HSP proteins is largely independent of 4E-BP1 regulated CAP-dependent translation providing a potential explanation for their maintained levels [43]–[45]. Release of HSPs induces macrophage infiltration, inflammation and phagocytosis of the dying cells. Consistent with our previous observation of macrophage infiltration of the bone marrow of everolimus treated leukemic mice (Figure S6) [14].

Cell death induced by mTOR inhibition in ALL cells is clearly a form of caspase-independent programmed cell death, but precise classification awaits clearer definitions of alternative cell death mechanisms. However, it is possible, and the authors suspect likely, that alternative cell death mechanisms form a spectrum of possible ways to die and therefore may not fit neatly into tightly defined classes.?It could be viewed that the caspase-independence of everolimus-induced cell death may provide an advantage in cells that may have become resistant to apoptosis, and mTOR inhibition can sensitize cells to apoptosis induced by other agents [17], [46]. Overall we can conclude that everolimus induces a caspase-independent cell death that is clearly not mitotic catastrophe, despite occurring in cells committed to mitosis, and that this cell death shares many features currently attributed to paraptosis.

## Supporting Information

Figure S1 **ALL cells treated with everolimus do not express β-galactosidase.**
(DOCX)

Figure S2 **ALL cell lines do not express p16.**
(DOCX)

Figure S3 **Cleavage of caspase-3 in NALM6 cells following exposure to doxorubicin.**
(DOCX)

Figure S4 **Everolimus induces PARP cleavage products consistent with necrotic cell death and activation of caspase-8 and -9.**
(DOCX)

Figure S5 **The JNK inhibitor SP600125 (JNKi) blocks everolimus driven phosphorylation of c-jun.**
(DOCX)

Figure S6 **Macrophage infiltration of bone marrow following everolimus treatment.**
(DOCX)

Table S1 **Details of Patient Samples.**
(DOCX)

Table S2 **Primers for qRT-PCR.**
(DOCX)

Table S3 **DAVID Analysis of Function by Keywords.**
(DOCX)
