# Supplementary material for: mTOR Inhibition by Everolimus in Childhood Acute Lymphoblastic Leukemia Induces Caspase-Independent Cell Death
Source: PLoS One. 2014 Jul 11;9(7):e102494. doi: 10.1371/journal.pone.0102494 (PMC4094511; doi:10.1371/journal.pone.0102494)
Supplement: Figure S1 — ALL cells treated with everolimus do not express β-galactosidase. (DOCX) [file pone.0102494.s001.docx]

**
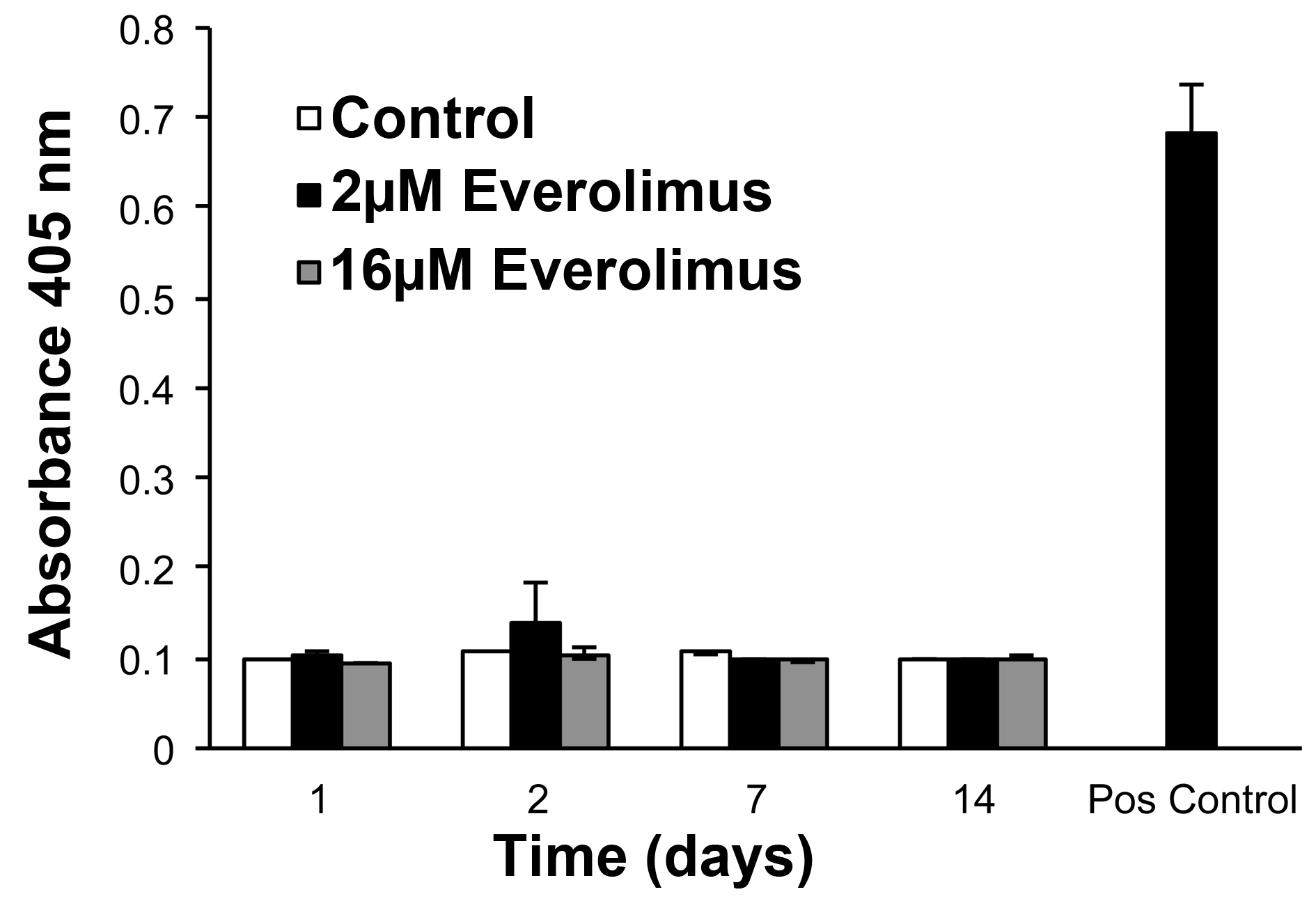
Figure S1. ALL cells treated with everolimus do not express β-galactosidase.** NALM6 cells were cultured with the indicated concentrations of everolimus for the specified time periods and assessed for the expression of β-galactosidase. The mean ± SD of duplicates is shown. The positive control consisted of human embryonic melanocytes that express p16 at normal levels.
