# Supplementary material for: mTOR Inhibition by Everolimus in Childhood Acute Lymphoblastic Leukemia Induces Caspase-Independent Cell Death
Source: PLoS One. 2014 Jul 11;9(7):e102494. doi: 10.1371/journal.pone.0102494 (PMC4094511; doi:10.1371/journal.pone.0102494)
Supplement: Figure S2 — ALL cell lines do not express p16. (DOCX) [file pone.0102494.s002.docx]

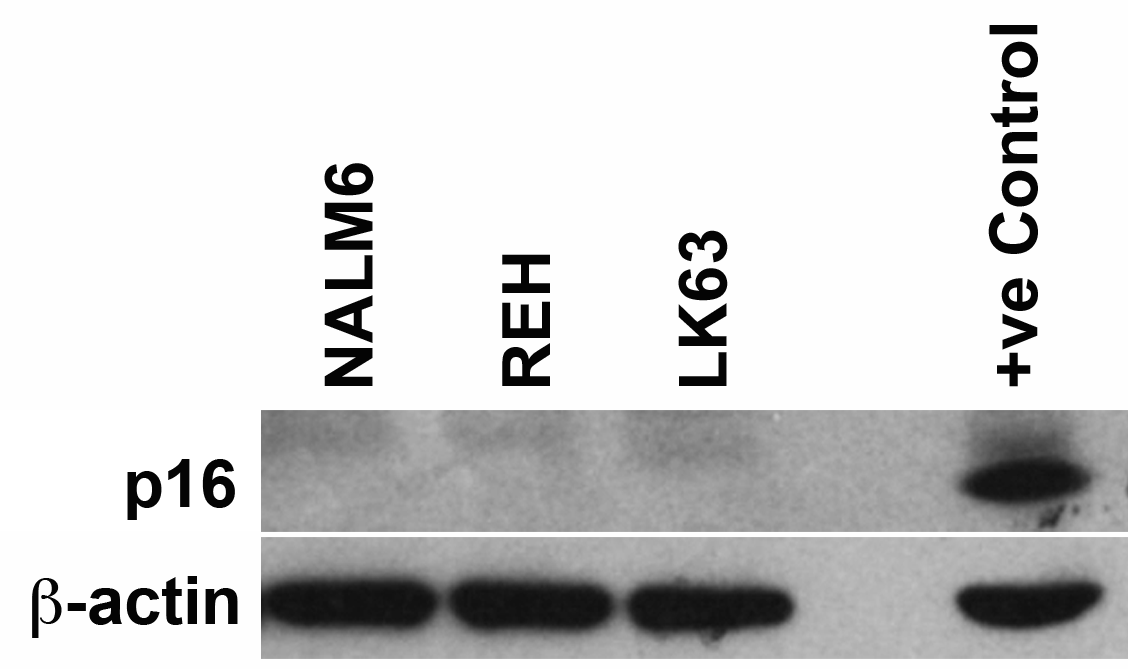


**Figure S2. ALL cell lines do not express p16.** Cell lysates were prepared from the indicated cell lines and analyzed by Western blotting for the expression of p16. Loading was assessed by probing for β-actin on the same membrane after stripping. The positive control consisted of the melanoma cell line NM179 which carries an intact INK4a/ARF locus.^44^
