# Supplementary material for: mTOR Inhibition by Everolimus in Childhood Acute Lymphoblastic Leukemia Induces Caspase-Independent Cell Death
Source: PLoS One. 2014 Jul 11;9(7):e102494. doi: 10.1371/journal.pone.0102494 (PMC4094511; doi:10.1371/journal.pone.0102494)
Supplement: Figure S3 — Cleavage of caspase-3 in NALM6 cells following exposure to doxorubicin. (DOCX) [file pone.0102494.s003.docx]

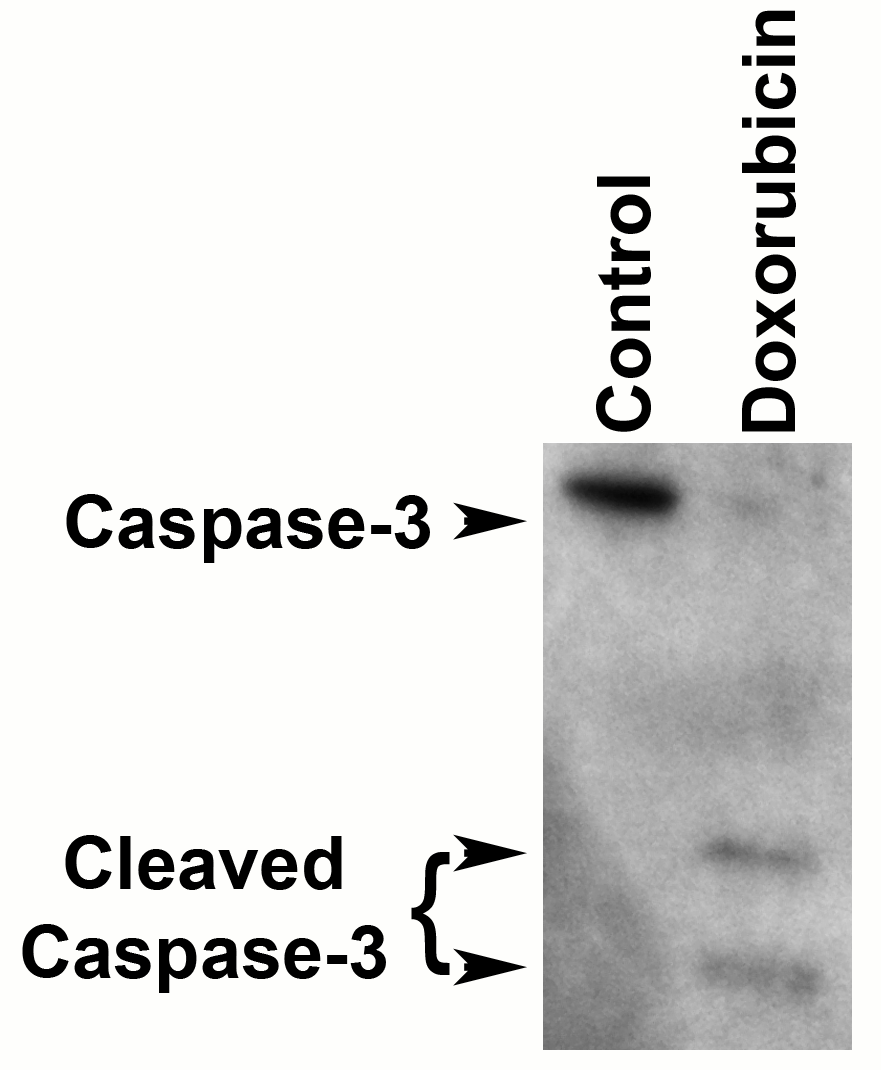


**Figure S3.** **Cleavage of caspase-3 in NALM6 cells following exposure to doxorubicin.** NALM6 cells were cultured for 6 h in the presence or absence (Control) of 10 μg/ml of doxorubicin, cell lysates prepared and probed for caspase-3 by western blotting.
