# Supplementary material for: mTOR Inhibition by Everolimus in Childhood Acute Lymphoblastic Leukemia Induces Caspase-Independent Cell Death
Source: PLoS One. 2014 Jul 11;9(7):e102494. doi: 10.1371/journal.pone.0102494 (PMC4094511; doi:10.1371/journal.pone.0102494)
Supplement: Figure S4 — Everolimus induces PARP cleavage products consistent with necrotic cell death and activation of caspase-8 and -9. (DOCX) [file pone.0102494.s004.docx]

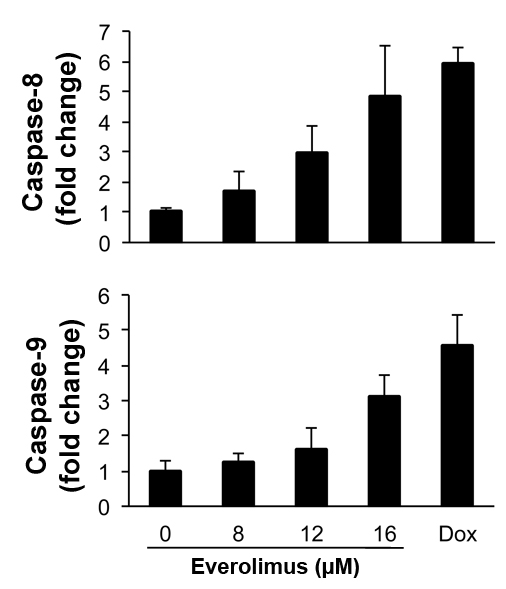


**Figure S4. Everolimus induces PARP cleavage products consistent with necrotic cell death and activation of caspase-8 and -9.** NALM6 cells were treated with the indicated concentrations of everolimus (Eve) or 1 μM doxorubicin for 6 h. Cell lysates were assessed for caspase-8 and -9 activity. The mean ± SD of 3 experiments is shown.
