# Supplementary material for: mTOR Inhibition by Everolimus in Childhood Acute Lymphoblastic Leukemia Induces Caspase-Independent Cell Death
Source: PLoS One. 2014 Jul 11;9(7):e102494. doi: 10.1371/journal.pone.0102494 (PMC4094511; doi:10.1371/journal.pone.0102494)
Supplement: Figure S5 — The JNK inhibitor SP600125 (JNKi) blocks everolimus driven phosphorylation of c-jun. (DOCX) [file pone.0102494.s005.docx]

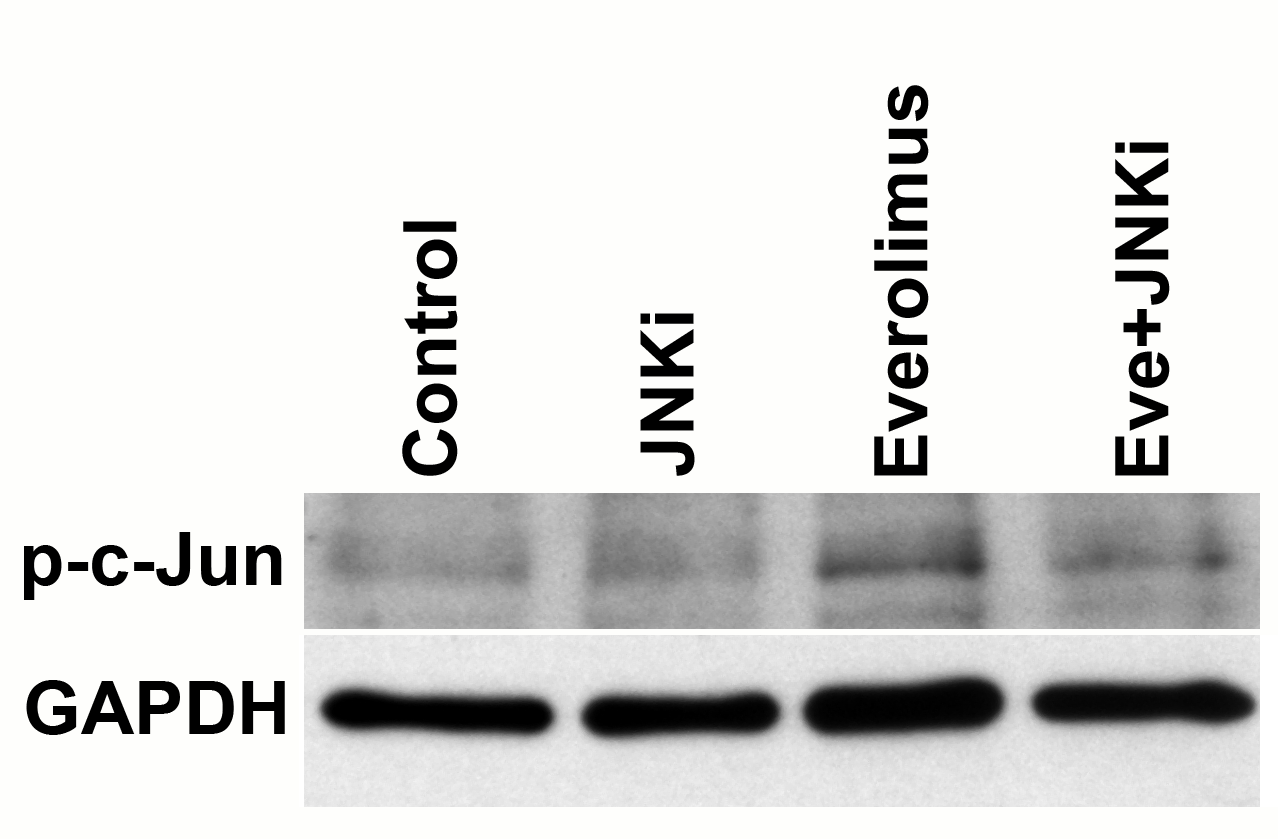


**Figure S5**. **The JNK inhibitor SP600125 (JNKi) blocks everolimus driven phosphorylation of c-jun.** NALM6 cells were treated with 5 μM SP600125 for 1 h prior to the addition of 16 μM everolimus. Cells were incubated for a further 10 mins prior to the preparation of cell lysates.
