# Supplementary material for: mTOR Inhibition by Everolimus in Childhood Acute Lymphoblastic Leukemia Induces Caspase-Independent Cell Death
Source: PLoS One. 2014 Jul 11;9(7):e102494. doi: 10.1371/journal.pone.0102494 (PMC4094511; doi:10.1371/journal.pone.0102494)
Supplement: Figure S6 — Macrophage infiltration of bone marrow following everolimus treatment. (DOCX) [file pone.0102494.s006.docx]

**
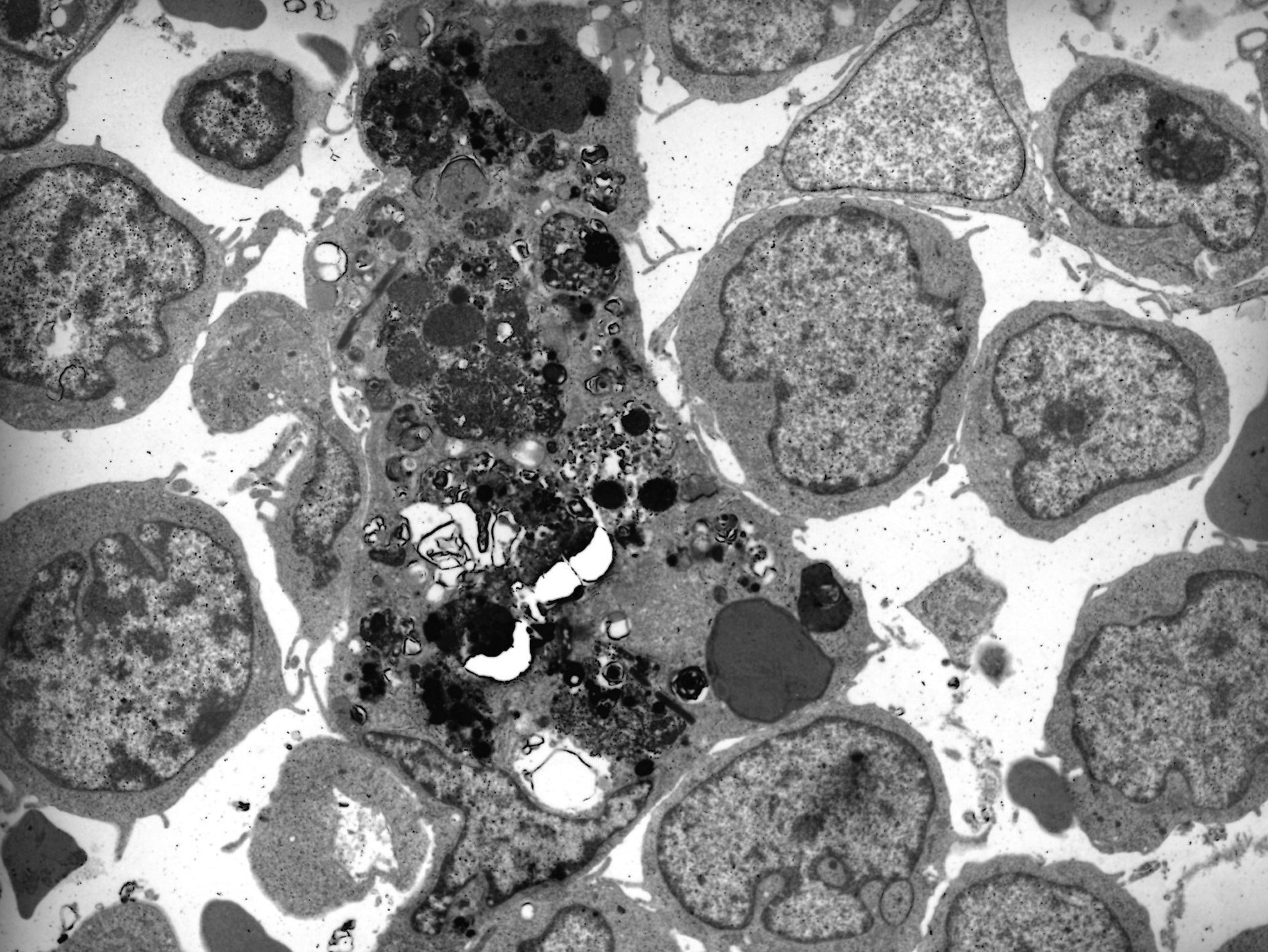
**

**Figure S6. Macrophage infiltration of bone marrow following everolimus treatment.** Electron microscopy of a mouse femur collected from an animal treated with 10 mg/kg everolimus as described.[^14^](#_ENREF_14)
