# Supplementary material for: mTOR Inhibition by Everolimus in Childhood Acute Lymphoblastic Leukemia Induces Caspase-Independent Cell Death
Source: PLoS One. 2014 Jul 11;9(7):e102494. doi: 10.1371/journal.pone.0102494 (PMC4094511; doi:10.1371/journal.pone.0102494)
Supplement: Table S1 — Details of Patient Samples. (DOCX) [file pone.0102494.s007.docx]

**Table S1. Details of Patient Samples**

| Patient ID | Age/Sex | Immunophenotype | Cytogenetics |
| --- | --- | --- | --- |
| 0407 | 45/M | CD34^-^ CD19^+^ CD10^+^ CD20^-^ | t (1;19)* |
| 1901 | 5m/F | CD34^+^ CD19^+^ CD10^-^ CD20^-^ | 46 XX, del(12)(p11.2p13)[6]/46, XX[14] |
| 2032 | 12/M | CD19^+^ CD10^+^ | 46 XY add (9)(p24), del(9)(p21), del(13)(q11q21), del(19) t(1;19)(q23;p13) |
| 2070 | 65/M | CD34^+^ CD19^+^ CD10^+^ CD20^+^ CD45^+^ | 45 XY t(9;22) (q34;q11.2) del(9) (p21) |
| 1345 | 5/F | CD34^-^CD19^+^ CD10^+^ CD20^+^ HLA-DR | 45 XX dup (1)(q42 q25), del (3) (q21),-9,del (9)p22,t(18;20)(q21;q13.1) |

*Complete karyotype not available but t(1:19) detected by PCR.
