# Supplementary material for: mTOR Inhibition by Everolimus in Childhood Acute Lymphoblastic Leukemia Induces Caspase-Independent Cell Death
Source: PLoS One. 2014 Jul 11;9(7):e102494. doi: 10.1371/journal.pone.0102494 (PMC4094511; doi:10.1371/journal.pone.0102494)
Supplement: Table S2 — Primers for qRT-PCR. (DOCX) [file pone.0102494.s008.docx]

**Table S2. Primers for qRT-PCR**

| Gene ID | Forward | Reverse |
| --- | --- | --- |
| *GADD153* | AAGTGGCTACTGACTACCCTCT | AAGCCTTCCCCCTGCGTAT |
| *GADD34* | ATGTATGGTGAGCGAGAGGC | GCAGTGTCCTTATCAGAAGGC |
| *TRB3* | CTGCCCGCTGTCTGGTTC | GGGCATCGGGTCCTGTCG |
| *ATF4* | CCACTAGGTACCGCCAGAAG | GCCTTGCGGACCTCTTCTAT |
| *ATF6a* | CACAGCTCCCTAATCACGTGG | ACTGGGCTATTCGCTGAAGG |
| *ATF6b* | CAGCCATCAGCCACAACAAG | GGCATCACCAGGGACATCTT |
| *PERK* | GCCTTGGATCCTGAAAATCA | GTCTTGGTCCCACTGGAAGA |
| *GAPDH* | ACGCATTTGGTCGTATTGGG | TGATTTTGGAGGGATCTCGC |
