# Supplementary material for: mTOR Inhibition by Everolimus in Childhood Acute Lymphoblastic Leukemia Induces Caspase-Independent Cell Death
Source: PLoS One. 2014 Jul 11;9(7):e102494. doi: 10.1371/journal.pone.0102494 (PMC4094511; doi:10.1371/journal.pone.0102494)
Supplement: Table S3 — DAVID Analysis of Function by Keywords. (DOCX) [file pone.0102494.s009.docx]

**Table S3. DAVID Analysis of Function by Keywords**

| **Term** | **Count** | **%** | **P Value^a^** | **Fold Enrichment** |
| --- | --- | --- | --- | --- |
| Acetylation | 102 | 40.0 | 4.20E-23 | 2.9 |
| Chaperone | 18 | 7.1 | 4.85E-09 | 8.7 |
| Molecular chaperone | 7 | 2.7 | 8.03E-07 | 40.9 |
| Stress response | 10 | 3.9 | 1.51E-05 | 11.5 |
| Phosphoprotein | 134 | 52.5 | 4.92E-05 | 1.4 |
| mRNA processing | 16 | 6.3 | 9.87E-05 | 4.7 |
| Mitochondrion | 29 | 11.4 | 2.22E-04 | 2.7 |
| Isopeptide bond | 16 | 6.3 | 8.66E-04 | 3.8 |
| Transit peptide | 19 | 7.5 | 0.002 | 3.0 |
| mRNA splicing | 12 | 4.7 | 0.003 | 4.4 |
| Cytoplasm | 68 | 26.7 | 0.004 | 1.6 |

a Benjamini Corrected for Multiple Comparisons. Count – number of genes in the dataset. % - the percentage of genes in the dataset.
